# Mortality by causes in HIV-infected adults: comparison with the general population

**DOI:** 10.1186/1471-2458-11-300

**Published:** 2011-05-11

**Authors:** Pablo Aldaz, Conchi Moreno-Iribas, Nerea Egüés, Fátima Irisarri, Yugo Floristan, Julio Sola-Boneta, Víctor Martínez-Artola, Mirian Sagredo, Jesús Castilla

**Affiliations:** 1 Centro de Salud de San Juan, Servicio Navarro de Salud, Avda Barañain 26, 31008 Pamplona, Spain; 2 Grupo de Prevención de Enfermedades Infecciosas, SEMFYC, Spain; 3 Instituto de Salud Pública de Navarra, Leyre 15, 31003 Pamplona, Spain; 4 CIBER de Epidemiología y Salud Pública, Spain; 5 Complejo Hospitalario de Navarra, Irunlarrea 2, 31008 Pamplona, Spain; 6 Red de Investigación en Sida (RIS), Spain

## Abstract

**Background:**

We compared mortality by cause of death in HIV-infected adults in the era of combined antiretroviral therapy with mortality in the general population in the same age and sex groups.

**Methods:**

Mortality by cause of death was analyzed for the period 1999-2006 in the cohort of persons aged 20-59 years diagnosed with HIV infection and residing in Navarre (Spain). This was compared with mortality from the same causes in the general population of the same age and sex using standardized mortality ratios (SMR).

**Results:**

There were 210 deaths among 1145 persons diagnosed with HIV (29.5 per 1000 person-years). About 50% of these deaths were from AIDS. Persons diagnosed with HIV infection had exceeded all-cause mortality (SMR 14.0, 95% CI 12.2 to 16.1) and non-AIDS mortality (SMR 6.9, 5.7 to 8.5). The analysis showed excess mortality from hepatic disease (SMR 69.0, 48.1 to 78.6), drug overdose or addiction (SMR 46.0, 29.2 to 69.0), suicide (SMR 9.6, 3.8 to 19.7), cancer (SMR 3.2, 1.8 to 5.1) and cardiovascular disease (SMR 3.1, 1.3 to 6.1). Mortality in HIV-infected intravenous drug users did not change significantly between the periods 1999-2002 and 2003-2006, but it declined by 56% in non-injecting drug users (*P *= 0.007).

**Conclusions:**

Persons with HIV infection continue to have considerable excess mortality despite the availability of effective antiretroviral treatments. However, excess mortality in the HIV patients has declined since these treatments were introduced, especially in persons without a history of intravenous drug use.

## Background

The expansion of combined antiretroviral treatments in the developed countries has been followed by substantial reductions in the incidence of AIDS-defining conditions and mortality among HIV-infected persons [1,2]. With these treatments and in the absence of other risk factors, mortality in HIV-infected persons approaches that of similarly aged persons with chronic diseases and is becoming closer to mortality in the general population of a similar age [3-5].

Mortality in HIV-infected persons depends on the duration of infection, age at the time of seroconversion and the effectiveness of the antiretroviral treatment [6]; this, in turn, depends on when treatment was started, whether previous treatment responses have been suboptimal, and the presence of coinfections [7,8]. Combined antiretroviral treatments were introduced when many countries had a considerable number of HIV- infection with long evolution of the disease and other comorbidities. These factors have meant that the real impact of these treatments on mortality has been less than what could be reached under ideal conditions. Various studies have related these treatments with substantial reductions in mortality from AIDS-defining conditions, but mortality from other causes may decrease to a lesser extent or may remain stable [8-10].

Many studies evaluating the effect of combined antiretroviral treatments on mortality in HIV-infected persons have been done in cohorts of patients during clinical follow-up [11], or who were being followed up regularly in HIV clinics [3,4,12,13], or who were beginning to receive combined antiretroviral treatment [14,15]. However, such studies may under-represent persons who died before the beginning medical follow-up, do not have regular check-ups, are difficult to recruit, or died from causes unrelated to HIV infection. Accordingly, such studies may describe scenarios that could be overly optimistic and not representative of the entire population of people living with HIV.

The objective of this study was to analyze total mortality and mortality by cause of death in a population-based cohort of adults diagnosed with HIV during a period of wide availability of combined antiretroviral treatments and to compare it with mortality in the general population of the same age and sex and living in the same region.

## Methods

This study analyzed the population-based cohort of persons residing in the region of Navarre (approximately 600,000 inhabitants), Spain, with a diagnosis of HIV infection confirmed by Western blot. The information was obtained from the HIV epidemiological surveillance system that was launched in Navarre in 1991. All HIV-infected patients diagnosed by laboratories of the region were incorporated retrospectively, followed by a continuous active search of all new laboratory-confirmed cases and patients attended in clinical centers [16,17]. Information on AIDS diagnoses was completed by reviewing the AIDS case register [16], and information on vital status was completed by reviewing the regional mortality records [18]. These data sources include AIDS diagnoses and deaths among Navarre residents occurred both within and outside the region that are reported at national level. To rule out the possibility of undetected HIV cases, AIDS diagnoses or deaths outside the region, additional searches were made in the dataset of hospital discharges, in the national AIDS registry, and in the national death index of Spain [19] (Table 1). All individuals who maintained their residence in Navarre and who were not shown as dead in any of these sources were considered to be alive at the end of the follow-up.

**Table 1:** Information sources used

Sources of information	Use
Epidemiological surveillance system of cases diagnosed with HIV-infection: active search of cases in records of laboratories and clinicians	Main source of HIV-infected cases
Hospital discharges database	Complementary source for quality control of HIV-infected cases
Regional AIDS-register	Main source of cases with AIDS-defining conditions
National AIDS-register	Complementary source for quality control of AIDS cases
Regional mortality register	Main source for deaths
National death index	Complementary source for quality control of deaths

Most HIV infections in Navarre have occurred in intravenous drug users, although sexual transmission has predominated in recent years. The entire population of the region has easy and free access to medical care and to the HIV test. Combined antiretroviral treatments are free and have been available since 1996 in accordance with internationally accepted treatment protocols [17].

The present analysis was limited to persons aged 20-59 years with stable residency in Navarre during the study period. The follow-up starting date was considered to be the date when HIV infection was diagnosed, the date when the person reached 20 years of age or 1 January 1999, whichever was latest. Follow-up of subjects was ceased at death or on 31 December 2006 for those who survived to that date. We calculated the mortality rates by cause of death, taking the number of person-years (PY) of follow-up as the denominator.

The reference population consisted of persons aged 20-59 years residing in Navarre according to the census data at the beginning of each study year, minus the number of person-years of follow-up corresponding to persons diagnosed with HIV infection. Deaths in the reference population were obtained from the regional mortality registry, and the number of deaths occurred in persons diagnosed with HIV were subtracted from it. The primary cause of death according to the International Classification of Diseases 10th edition was also obtained from this mortality registry [20].

The causes of death were grouped into the following categories: AIDS or HIV infection (B20-B24, R75), hepatic disease (B15-B19, B70, K73, K74, K769), non-AIDS defining cancers (C00-D48), drug addiction and overdose (F11, F16, F18, F19, X41, X42, X44, X45), cardiovascular disease (I00-I99), suicide (X60-X84), and other external causes (V01-Y89, excluding codes included in the preceding categories). Death certificates were reviewed from deaths coded as B20.3 and B23.8, and when the cause of death was liver disease or cirrhosis it was reclassified in the category of liver diseases rather than AIDS [20]. Deaths from acute pulmonary edema or other non-specific causes in parenteral drug users were reviewed taking the forensic report into account.

Mortality in persons diagnosed with HIV infection was compared with mortality in the rest of the population by calculating standardized mortality ratios (SMR) adjusted for sex and 5-year age groups; the 95% confidence intervals were obtained by applying the Poisson distribution. The analyses were stratified by sex and history of intravenous drug use to rule out the influence of such variables in the mortality comparisons.

Mortality in the HIV-infected cohort during the period of 1999-2002 was compared with that of during the period of 2003-2006 using Cox regression models, with age as the underlying time variable. Exit time was defined as the date of death or the end of the period, whichever came first.

The χ^2 ^test was used to compare proportions and values of *P *< 0.05 were considered to be significant.

## Results

### Characteristics of the HIV-infected subjects who died

On 1 January 1999, there were 879 individuals aged 20-59 years in follow-up in Navarre who had been diagnosed with HIV infection, and 266 new cases were added by the end of 2006. Of all these cases, 68% were men, 63% had a history of intravenous drug use, and 35% had a previous diagnosis of AIDS or were diagnosed with AIDS during follow-up. Between 1999 and 2006, there were 210 deaths among individuals in follow-up (29.5 per 1000 person-years) with a higher rate of deaths in men (34.2 per 1000 PY), in intravenous drug users (33.5 per 1000 PY), and in persons previously diagnosed with AIDS (67.1 *vs*. 15.2 per 1000 PY, *P *< 0.001). Over half of those included in the cohort had been diagnosed with HIV infection at least 5 years before, and mortality was higher in this group (Table 2).

**Table 2:** Characteristics of persons included in the cohort of HIV-infected subjects, and number and rate of deaths according to these characteristics; Navarre, Spain, 1999-2006

	HIV-infected subjects			Deaths		*P-value *
		
	N	%	Person-years	N	Rate per 1000 person-years	
Sex						<0.001
Male	781	68	4734	162	34.2	
Female	364	32	2379	48	20.2	
						
Risk categories						<0.001
Injecting drug users	721	63	4839	162	33.5	
Homo-/bisexual men	85	7	478	5	10.5	
Heterosexual	263	23	1328	34	25.6	
Other/unknown	76	7	877	9	10.3	
						
Country of origin						0.398
Spain	1024	89	6655	200	30.0	
Other	121	11	458	10	21.8	
						
AIDS-defining condition						<0.001
Yes	402	35	1967	132	67.1	
No	743	65	5146	78	15.2	
						
Year of HIV diagnosis						<0.001
1990 or before	307	27	1744	76	43.6	
1991-1995	354	31	1779	75	42.2	
1996-1999	242	21	1787	35	19.6	
2000-2006	242	21	1802	24	13.3	
						
Total	1145	100	7113	210	29.5	

*P value *for comparison of rates within categories.

50.5% of deaths were due to AIDS-defining conditions, 17.1% due to liver diseases, 11.0% due to drug overdose or addiction, 8.1% due to non-AIDS-defining cancers, 3.8% due to cardiovascular disease and 3.3% due to suicides.

The median time from HIV diagnosis to death was 11.4 years. About 5.2% (n = 11) of he deaths occurred in the first 3 months after the HIV diagnosis. This percentage was higher in parenteral drug users (16.3% *vs*. 1.9%, *P *< 0.001). Ten of these deaths were from AIDS-defining conditions and one from hepatic disease.

### Comparison with the general population

Mortality among persons diagnosed with HIV was 14 times higher than mortality in the general population of the same sex and age groups (SMR, 14.0; 95% CI, 12.2 to 16.1); after excluding deaths from AIDS, high mortality in individuals with HIV persisted and was 6.9 times higher (5.7 to 8.5). Such high mortality in persons diagnosed with HIV was mainly from the liver disease (SMR 69.0, 48.1 to 78.6), drug overdose or addiction (SMR 46.0, 29.2 to 69.0) and suicide (SMR 9.6, 3.8 to 19.7) and, in lesser measure, from non-AIDS defining cancers (SMR 3.2, 1.8 to 5.1), cardiovascular disease (SMR 3.1, 1.3 to 6.1) and other causes (SMR 5.1, 2.3 to 9.7). Excess mortality in persons diagnosed with HIV was more pronounced in those who had a history of intravenous drug use (SMR 17.7, 15.2 to 20.8) than in those who did not (SMR 8.2, 6.0 to 11.0). Among the former group, there was notably increased mortality from causes other than AIDS (SMR 9.6, 7.7 to 12.0); especially from liver disease (SMR 96.6, 65.2 to 138.1) and from drug overdose or addiction (SMR 63.4, 40.2 to 95.1) (Table 3).

**Table 3:** Standardized mortality ratio by cause of death, injecting-drug status and sex in HIV-infected subjects compared with persons in the general population not diagnosed with HIV infection; Navarre, Spain, 1999-2006

	Observed deaths	Expected deaths	Standardized mortality ratio	(95% CI)
**All HIV-infected subjects**				
All-cause deaths	210	15.0	14.0	(12.2-16.1)
Non-AIDS deaths	104	15.0	6.9	(5.7-8.5)
Liver disease	36	0.5	69.0	(48.1-78.6)
Non-AIDS defining cancer	17	5.4	3.2	(1.8-5.1)
Drug overdoses or addiction	23	0.5	46.0	(29.2-69.0)
Cardiovascular disease	8	2.6	3.1	(1.3-6.1)
Suicide	7	0.7	9.6	(3.8-19.7)
Other external causes	4	3.0	1.3	(0.4-3.4)
All other causes	9	1.8	5.1	(2.3-9.7)
				
**Injecting drug users**				
All-cause deaths	162	9.1	17.7	(15.2-20.8)
Non-AIDS deaths	88	9.1	9.6	(7.7-12.0)
Liver disease	30	0.3	96.6	(65.2-138.1)
Non-AIDS defining cancer	12	3.0	4.0	(2.1-7.0)
Drug overdoses or addiction	23	0.4	63.4	(40.2-95.1)
Cardiovascular disease	6	1.5	4.0	(1.5-8.6)
Suicide	5	0.5	10.8	(3.5-25.3)
Other external causes	4	2.0	2.0	(0.5-5.1)
All other causes	8	1.1	7.3	(3.2-14.4)
				
**Non-injecting drug users**				
All-cause deaths	48	5.9	8.2	(6.0-11.0)
Non-AIDS deaths	16	5.9	2.7	(1.6-4.4)
Liver disease	6	0.2	28.4	(10.4-62.0)
Non-AIDS defining cancer	5	2.3	2.1	(0.7-5.0)
Drug overdoses or addiction	0	0.1	0	
Cardiovascular disease	2	1.1	1.8	(0.2-6.6)
Suicide	2	0.3	7.4	(0.89-26.6)
Other external causes	0	1.0	0	
All other causes	1	0.7	1.5	(0.04-8.2)
				
**Male**				
All-cause deaths	162	12.5	12.9	(11.0-15.1)
Non-AIDS deaths	78	12.5	6.2	(4.9-7.9)
Liver disease	25	0.5	54.1	(35.0-80.1)
Non-AIDS defining cancer	13	4.1	3.1	(1.7-5.4)
Drug overdoses or addiction	20	0.5	43.3	(26.5-66.7)
Cardiovascular disease	6	2.4	2.5	(0.9-5.6)
Suicide	4	0.5	7.3	(2.0-18.7)
Other external causes	3	2.7	1.1	(0.2-3.3)
All other causes	7	1.4	5.0	(2.0-10.2)
				
**Female**				
All-cause deaths	48	2.5	19.5	(14.2-26.1)
Non-AIDS deaths	26	2.5	10.6	(6.9-15.5)
Liver disease	11	0.1	183.3	(91.5-328.1)
Non-AIDS defining cancer	4	1.2	3.3	(0.9-8.3)
Drug overdoses or addiction	3	0.04	78.5	(16.2-229.3)
Cardiovascular disease	2	0.2	8.0	(1.0-29.0)
Suicide	3	0.2	16.4	(3.4-47.8)
Other external causes	1	0.3	2.9	(0.1-16.4)
All other causes	2	0.4	5.5	(0.7-20.0)

Among non-injecting drug users, mortality from causes other than AIDS (SMR 2.7, 1.6 to 4.4) was still higher than the general population but less than that among the intravenous drug users. In non-injecting drug users the increase in mortality was again primarily due to liver diseases (SMR 28.4, 10.4 to 62.0) (Table 3).

Cox regression models were used to compare mortality in the cohort of HIV-infected persons between the periods of 1999-2002 and of 2003-2006, adjusted for sex, age, intravenous drug use and country of origin. AIDS mortality in HIV-infected persons decreased by 37% (adjusted hazard ratio [HR] 0.63; 95% CI 0.42-0.95; *P *= 0.026). Mortality in intravenous drug users remained high with a non-statistically significant change between the two periods (HR 0.85; 95% CI 0.60-1.19; *P *= 0.336), whereas mortality in non-injecting drug users decreased by 56% (HR 0.44; 95% CI 0.24-0.79; *P *= 0.007), with a 51% reduction in mortality caused by AIDS (HR 0.49; 95% CI 0.24-1.02; *P *= 0.057) and 67% reduction in mortality from causes other than AIDS (HR 0.33; 95% CI 0.11-0.96; *P *= 0.041) (Table 4).

**Table 4:** Comparison of mortality by cause of death in HIV-infected cohort in the periods 1999-2002 and 2003-2006; Navarre, Spain.

	Period 1999-2002		Period 2003-2006				
				
	Cases	Rate per 1000 PY	Cases	Rate per 1000 PY	Hazard ratio*	(95% CI)	*P-value *
**All HIV-infected subjects**							
All-cause deaths	115	32.6	95	26.5	0.72	(0.54-0.96)	0.024
AIDS deaths	62	17.6	44	12.3	0.63	(0.42-0.95)	0.026
Non-AIDS related deaths	53	15.0	51	14.2	0.82	(0.54-1.23)	0.325
Liver disease	14	4.0	22	6.1	1.34	(0.66-2.72)	0.420
Non-AIDS defining cancer	10	2.8	7	2.0	0.47	(0.17-1.30)	0.145
Drug overdoses or addiction	14	4.0	9	2.5	0.63	(0.26-1.53)	0.307
Cardiovascular disease	3	0.9	5	1.4	1.12	(0.25-5.06)	0.880
Suicide	3	0.9	4	1.1	1.26	(0.26-6.04)	0.776
Other external causes	3	0.9	1	0.3	0.21	(0.02-2.16)	0.190
All other causes	6	1.7	3	0.9	0.58	(0.13-2.50)	0.461
							
**Injecting drug users**							
All-cause deaths	85	33.5	77	33.5	0.85	(0.60-1.19)	0.336
AIDS deaths	43	16.9	31	13.5	0.68	(0.41-1.13)	0.132
Non-AIDS related deaths	42	16.5	46	20.2	1.02	(0.65-1.61)	0.933
							
**Non-injecting drug users**							
All-cause deaths	30	30.4	18	14.0	0.44	(0.24-0.79)	0.007
AIDS deaths	19	19.3	13	10.1	0.49	(0.24-1.02)	0.057
Non-AIDS related deaths	11	11.2	5	3.9	0.33	(0.11-0.96)	0.041

PY, person-years. CI, confidence interval.

*Hazard ratio obtained in Cox regression models with age as underlying time variable and adjusted for sex, country of origin (Spain or others) and injecting drug use.

## Discussion

Persons diagnosed with HIV infection had considerable excess mortality in comparison with the general population of the same age group and sex, despite wide availability of combined antiretroviral treatments. This excess mortality was largely due to deaths from AIDS-defining conditions, but other causes of death were also important, such as drug overdose or addiction, hepatic disease, non-AIDS-defining cancers, and cardiovascular disease. Excess mortality associated with HIV infection was observed in both men and women, and in persons with and without a history of injecting drug use.

Numerous studies have reported substantial reductions in mortality in HIV-infected persons after the introduction of combined antiretroviral treatments [21,22], the same as we found in Navarre [9]. Nonetheless, mortality in this group is still 14 times higher than in the general population after adjusting for sex and age group. Some studies have found somewhat lower excess mortality [23], but different results could be explained by the epidemiological characteristics of those infected, time of evolution of the infection, and whether or not persons who are not receiving antiretroviral treatment are included. Health authorities should be alert not only to those causes with relative excess mortality, but also to those that are responsible for the largest absolute number of deaths.

In the era of combined antiretroviral treatments, mortality among persons diagnosed with HIV has continued to decline, mainly in those without a history of injecting drug use [21], but is still a long way from reaching mortality levels similar to those in the general population [5].

We found that the weight of AIDS-defining diseases has decreased in favor of other causes, although they continue to be the leading cause of death in HIV-infected persons [24]. Possible explanations for this include delayed diagnosis of HIV infection, HIV-infected subjects who refuse to receive antiretroviral treatment, and low adherence to these treatments. Delayed diagnosis has been detected in 37% of cases of HIV-infection diagnosed in Spain [25]. People who are unaware of their infection probably have a higher risk of death than diagnosed subjects, thus earlier diagnosis would reduce the excess mortality. Some misclassification is also possible since doctors may be prone to certify deaths in HIV-infected subjects as due to AIDS; however, this is unlikely because classification of a death as due to AIDS requires the presence of an AIDS-defining condition.

Liver diseases were the second cause of death in persons living with HIV, which may be explained by coinfections with hepatitis B or C viruses, which share the same sexual and parenteral mechanisms of transmission as HIV, and by high levels of alcohol and drug use [26]. Of note is the high mortality associated with drug overdose or addiction [14,27], which could have previously been partly masked by AIDS mortality and may only now be coming to the fore due to improved survival in HIV infection [4]. Excess cardiovascular mortality has been related with some antiviral treatments [28], but recent studies suggest an effect of the infection itself [29]. Non-AIDS-defining cancers play a larger than expected role as a cause of death in HIV-infected persons [30], comprising 8% of deaths, a percentage close to that found in other studies [31]. Among the explanations offered for these types of cancer are HIV-induced immunosuppression and the high frequency of unhealthy habits like smoking [32]. Combined antiretroviral treatments may be reducing the mortality of both AIDS-defining and non-AIDS-defining cancers [33]. In agreement with other studies, we found a high mortality from suicide among HIV-infected persons [34]. Despite advances in the treatment of HIV infection and increased survival in these patients, their mortality rates remain higher than those in the general population [35]. Only by investigating the factors that determine these causes of death will we be able to act on them.

Our study provides a population-based view of mortality by causes of death in HIV-infected people, including cases and events that could be not detected in clinical cohorts. Therefore, we have not included some baseline variables such as antiretroviral treatments, viral load and CD4 cell count which are relevant from a clinical perspective.

Losses to follow-up are one of the main sources of bias in follow-up studies. We tried to reduce such losses in our study by searching for subjects in a variety of information sources in order to detect or rule out their death. AIDS-related deaths included those due to AIDS-defining conditions, including opportunistic infections, some cancers and tuberculosis, which are the most frequent but not exclusive causes of death in persons with HIV-infection. We partly overcame this problem of comparability with the general population by not including deaths from liver disease in HIV-infected persons as deaths from AIDS. However, it was not possible to compare persons with HIV and the general population with respect to deaths from AIDS-defining diseases. Our results are marked by the epidemiological pattern in the study area, which is characterized by a predominance of HIV infections acquired in relation with injecting drug use [17]. Thus, they may not be generalized to other areas with a different epidemiological pattern.

## Conclusions

In the era of combined antiretroviral treatments, we still find considerable excess mortality in persons living with HIV. Since these treatments were introduced, advances have continued to be made in reducing this excess mortality, especially in persons without a history of injecting drug use. Further reduction will require improvements in early diagnosis of HIV and compliance with treatment as well as special emphasis on measures to prevent cancer, cardiovascular disease, and the whole range of problems related with injecting drug use.

## List of abbreviations

HIV: human immunodeficiency virus; AIDS: acquired immunodeficiency syndrome; SMR: standard mortality ratio; PY: person-year; HR: hazard ratio.

## Competing interests

The authors declare that they have no competing interests.

## Authors' contributions

All the authors participated in the data preparation and analysis, and also contributed to and approved the final manuscript. Additionally, PA, JC and CMI designed the original cohort study, planned the statistical analysis and wrote the draft.

## Pre-publication history

The pre-publication history for this paper can be accessed here:

http://www.biomedcentral.com/1471-2458/11/300/prepub
